# First report of a member of the family Mermithidae parasitizing the sandhopper *Orchestoidea tuberculata* (Amphipoda, Talitridae) in Chile

**DOI:** 10.1016/j.ijppaw.2023.10.011

**Published:** 2023-10-31

**Authors:** Sara M. Rodríguez, Marcela Figueroa, Guillermo D’Elía, Mario George-Nascimento

**Affiliations:** a Departamento de Ecología, Facultad de Ciencias, Universidad Católica de la Santísima Concepción, Concepción, Chile; b Centro de Investigación en Recursos Naturales y Sustentabilidad (CIRENYS), Universidad Bernardo O'Higgins, Avenida Viel 1497, Santiago de Chile, Chile; c Instituto de Ciencias Marinas y Limnológicas, Facultad de Ciencias, Universidad Austral de Chile. Isla Teja s/n, Valdivia, Chile; d Instituto de Ciencias Ambientales y Evolutivas, Facultad de Ciencias, Universidad Austral de Chile, Valdivia, Chile; e Centro de Investigación en Biodiversidad y Ambientes Sustentables (CIBAS), Universidad Católica de la Santísima Concepción, Concepción, Chile

**Keywords:** Mermithids, Semiterrestrial amphipods, COI, 18S, Chile

## Abstract

Specimens of the sandhopper *Orchestoidea tuberculata* (Amphipoda; Talitridae) collected from sandy beaches in south-central Chile, were found to be parasitized by juvenile mermithids, constituting the first record of a mermithid infecting a marine amphipod in Chile. A morphological description of juveniles is provided. Sequence analyses based on mitochondrial COI and nuclear 18S rDNA of the mermithids showed extremely low genetic variation. Phylogenetic analyses indicate that the mermithid is more closely related to *Hexamermis agrotis*, which parasitize Coleoptera, than to *Thaumamermis zealandica*, which parasitizes New Zealand confamilial talitrid amphipods.

## Highlights

• First record of a mermithid infecting a semiterrestrial amphipod in Chile.
• Sandhopper *Orchestoidea tuberculata* were found parasitized by juvenile mermithids.
• Sequences analyzed by COI and 18S showed extremely low genetic variation.
• Phylogenetic analyses indicate that the mermithid is close to parasites that inhabit in Coleoptera than those which parasitized to confamilial talitrids.

## Graphical abstract

**Figure:**
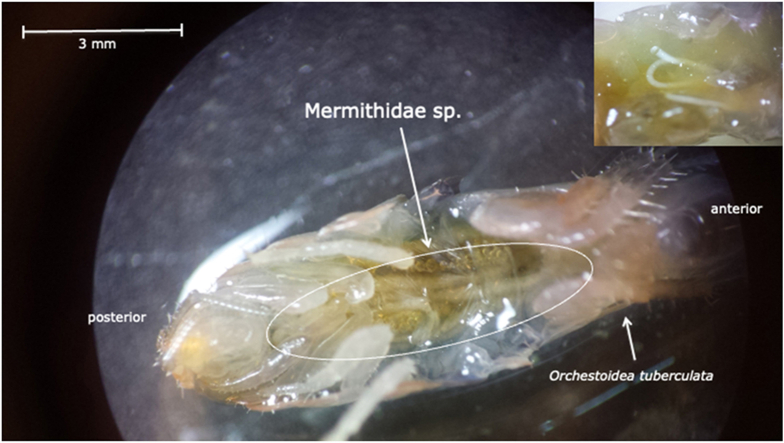

## 1 Introduction

Mermithidae Braun, 1883 is a family of nematodes that are obligate endoparasites of terrestrial and aquatic arthropods, especially insects. Their life cycle includes a parasitic and a free-living stages. The larval stages infect invertebrate hosts, develop within them to the juvenile stage is reached and then, they kill their hosts by breaking through the exoskeleton to mature in the external environment as free living nematodes (Nickle, 1972; Poinar, 1981). Mermithids parasitizing mosquitoes are the most studied group, as they can be used as biological agents to control host populations (Martinet et al., 2023); on the contrary, mermithids that parasite semiterrestrial crustaceans have been poorly reported despite their presence on almost all the coasts of the world (Väinölä et al., 2008; Pérez-Schultheiss, 2009). Mermithids of the genera *Agamomermis*, *Limnomermis*, and *Pseudomermis* have been reported infecting aquatic amphipods (Poinar et al., 2002). For instance, juveniles of *Thaumamermis zealandica* were found infecting the intertidal marine amphipod *Bellorchestia quoyana* from the coast of New Zealand (Poinar et al., 2002). In Chile, mermithids have been reported only from insects such as *Procalus mutans* and *P. reduplicatus* (family Chrysomelidae; Jerez and Centella, 1996) and *Tropisternus setiger* (family Hydrophilidae, Jerez and Moroni, 2006). Here we present the first Chilean report of a mermithid parasitizing an amphipod, the intertidal marine talitrid *Orchestoidea tuberculata* Nicolet, 1849 (Amphipoda, Talitridae) collected at sandy beaches of two locations in south–central coast of Chile.

## 2 Materials and methods

**Fig. 1:**
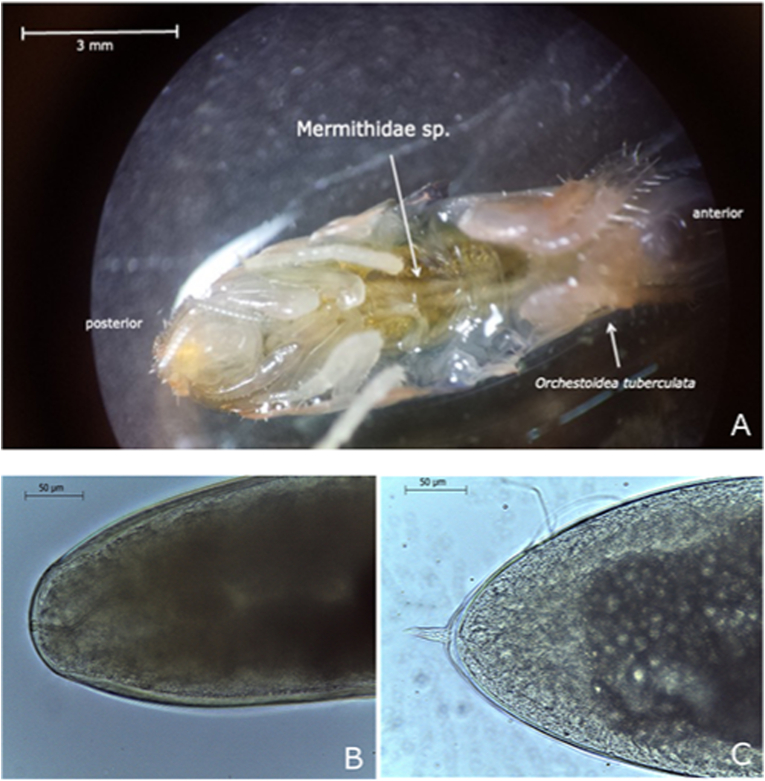
The mermithid nematode and the host *Orchestoidea tuberculata* sampled in this study. A. Ventral view of *Orchestoidea tuberculata* parasitized by a juvenile mermithid. B. Head of the nematode, C. Tail showing straight spur of the nematode.

Fifty-six and 30 specimens of *Orchestoidea tuberculata* were collected by hand under stranded kelp *Durvillaea antarctica* (Chamisso) Hariot in autumn 2019 at Ramuntcho beach (36.751° S; 73.184° W), BíoBío Region, and at Curiñanco beach (39.750° S, 73.392° W), Los Ríos Region, south-central, Chile, respectively. Amphipods were collected in plastic bags, transferred to the laboratory and frozen. Later, amphipods were examined under a stereomicroscope in the laboratory and nematodes were removed from them (Fig. 1A). The morphology of the head and tail of the worms was observed under a light microscope, and worms were measured using Leica Microsystems Framework. Finally, after observation, nematodes were fixed with ethanol 90% (Fig. 1B and C). Parasitological descriptors as prevalence and intensity were calculated as in Bush et al. (1997).

Genetic comparison and phylogenetic analyses were based on a fragment of 625 bp of the mitochondrial cytochrome oxidase I (COI) gene and a 755 bp fragment of the nuclear 18S rDNA gene. DNA of parasites was extracted from whole individuals collected at both beaches. Three and one specimens from Ramuntcho and Curiñanco, respectively, were sequenced for COI, and four and two specimens from Ramuntcho and Curiñanco, respectively were sequenced for 18S (Supplementary Table 1). The COI gene was amplified using the primers described by Folmer et al. (1994). PCR amplification of COI was carried out as performed by Rodríguez et al. (2017). The 18S rDNA gene was amplified using the primers and protocol described by Rodríguez et al. (2022). Amplicons were sequenced using an external sequencing service (Macrogen Inc., Seoul, South Korea). New DNA sequences were edited using Codon-Code (Codon Code Aligner, Dedham, Massachusetts, USA) and deposited in GenBank (COI=OP328787–OP328790; 18S rDNA = OR177418–OR177423).

Sequences of mermithids were integrated into two matrices, one per gene, with sequences downloaded from Genbank. Genes were analyzed separately due to the large difference on the taxonomic coverage available for each gene. Sampling included all available sequences belonging to species of the family Mermithidae (Table S1) as a way to assess the phylogenetic position of the Chilean genetic variants and then to try to molecularly identify the Chilean specimens. The matrix for COI was formed by sequences of five species of four genera and of four mermithids from this study (Table S1). The matrix for 18S was formed by sequences of 19 species of 17 genera, 10 sequences of unidentified mermithids, and of six mermithid specimens from this study (Table S1). Each gene matrix was aligned using MAFTT v.7 (Katoh and Standley, 2013), allowing the program to choose the alignment strategy (COI = L-ins-i; 18S = FFT–NS–i). The K3Pu + F + I (log-likelihood: 3093.749) and TIM2+F + I + G4 (log-likelihood: 10121.483) substitution models were chosen according by BIC for COI and 18S, respectively, using IQ-Tree v1.6.12. Gene trees were obtained using maximum-likelihood as implemented in IQ-Tree v1.6.12 (Trifinoupoulus et al., 2016; Kalyaanamoorthy et al., 2017). Nodal support was evaluated using two approaches: the aBayes test (Anisimova et al., 2011) and the ultra-fast bootstrap procedure with 1000 replicates (Hoang et al., 2018). Finally, the observed genetic *p*-distances (p) between haplotype and sample pairs were calculated in MEGA 7 (Tamura et al., 2013).

## 3 Results

Prevalence was 66 % at Ramuntcho beach (n = 56) and 26% at Curiñanco beach (n = 30). Infection intensity was 1 worm/host in both beaches. All the mermithids found were juveniles, as they were removed from their already frozen hosts (see Materials and methods) and no gonads were observed. They had a medium to large, white, narrow, and filiform bodies, measuring between 49 mm and 197 mm in length (*n* = 10; *mean* = 147 mm, *SD* ± 8 mm) and between 0.30 mm and 0.45 mm in width (*mean* = 0.36 mm, *SD* ± 0.04 mm), respectively. The head was homocephalic, the mouth cephalic and centered and had 6 cephalic papillae. Nerve ring was not clearly observed. Trophosoma prominent; tail rounded with a straight spur (37,52 μm) in the middle of the posterior part (Fig. 1A–C).

**Fig. 2:**
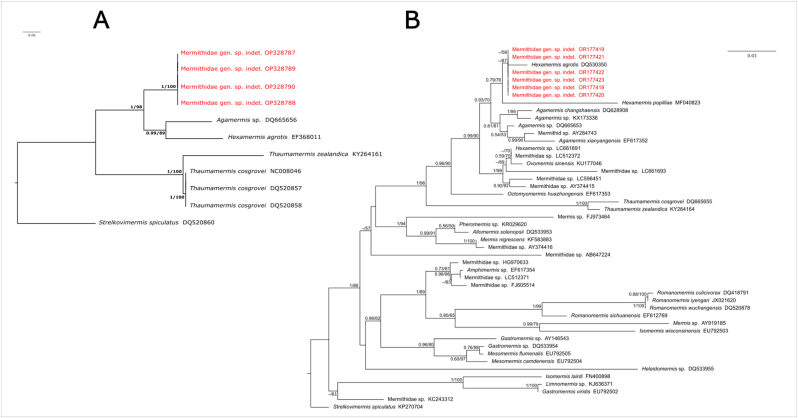
Genealogical relationships of haplotypes based on partial sequences of the mitochondrial cytochrome oxidase I (COI) **(A)** and 18S ribosomal DNA **(B)** gene sequences of specimens of the family Mermithidae recovered in a Bayesian inference analysis. Numbers next to nodes refer to support values. Bayesian posterior probability values are shown left of the diagonal. Bootstrap support values found in the Maximum Likelihood analysis are shown right of the diagonal. Accession numbers from GenBank are included in the terminal labels. Sequences in red were generated in this study. (For interpretation of the references to colour in this figure legend, the reader is referred to the Web version of this article.)

No variation was found among the COI sequences of our nematode specimens. Both phylogenetic trees gathered via ML and BI for COI were congruent (PP = 1; BS = 100; Fig. 2A). The clade formed by the Chilean Mermithidae gen. sp. indet. (OP328787-328790) is sister to clade formed by *Agamermis* sp. (DQ665656) and *Hexamermis agrotis* (EF368011)*,* which was highly supported (PP = 1; BS = 98). This clade formed by Mermithidae gen. sp. indet., *Agamermis* sp. and *H. agrotis* is sister to a clade formed by two species of *Thaumamermis.* Within the latter, *T. zealandica* is sister to a clade formed by samples of *T. cosgrovei*, which was highly supported (PP = 1; BS = 100) in this tree. The average observed genetic *p*-distance between Mermithidae gen. sp. indet. (OP328787-328790) and those of the clade *Agamermis* sp. (DQ665656) and *H. agrotis* (EF368011) is 18% (no variation was observed in these estimates as no variation was found in the sample of the Chilean Mermithidae gen. sp. indet. and those of *Agamermis* sp. and *H. agrotis* are composed by a single sequence). The observed genetic *p*-distance between the sequences of the Chilean Mermithidae gen. sp. indet., the sequence of *T. zealandica* and those of the clade formed by *T. cosgrovei* is 29% and 24%, respectively (similarly, the values show no variation as no variation is seem in the samples of the Chilean Mermithidae gen. sp. indet. and *T. cosgrovei* and the sample of *T. zealandica* is composed by a single sequence). Finally, the average genetic *p*-distance between the sequences of Mermithidae gen. sp. indet. and the sample of *Strelkovimermis spiculatus* is 31%. These results indicate that the interpecific values of *p*-distance ranged from 18 to 29%, which are much higher values than the one observed for the comparisons of the Chilean mermithid sequences (0%).

The resulting ML and BI trees for the 18S rDNA are congruent (Fig. 2B). Several genera, such as *Hexamermis*, *Mermis, Gastromermis* and *Mesomermis,* appear as non-monophyletic. The topologies show that the sequences of the Chilean Mermithidae gen. sp. indet. form a clade with that of *Hexamermis agrotis*, which has only moderate support in the ML analysis (PP < 0.50; BS = 87). This clade is sister to *H. popilliae* (PP = 0.79; BS = 76); the clade formed by the Chilean samples and *Hexamermis* is sister (PP = 0.93; BS = 70) to the clade formed by species of the genus *Agamermis* and an unidentified mermithid (PP = 0.81; BS = 81) in this tree. As such, the Chilean mermithid appears to be distantly related to *Thaumamermis* spp. (Fig. 2B). The values of *p*-distance among sequences of 18S rDNA ranged 3–11%, which is higher than those of Chilean Mermithidae gen. sp. indet (OR177418-177423) (0%).

## 4 Discussion

This is the first record of a mermithid infecting a marine amphipod in Chile, and the second record of a mermithid parasitizing a talitrid species at sandy beaches of the world. Although the morphological identification of nematodes (including mermithids) must be carried out in adults, which in mermithids correspond to a free-living stage, molecular analyses carried out on juvenile parasites showed that they may correspond to a previously unreported species. Most studies on mermithids have been carried out on juvenile stage; therefore, morphological characters are not well defined, preventing a reliable species identification on the parasites (Nickle, 1972; Stubbins et al., 2016; Yoshino and Waki, 2021). In fact, the morphological characters of the mermithid studied here differ from those of juvenile *Thaumamermis* specimens of New Zealand marine amphipods (*n =* 1). For example, the position of the spur in *Thaumamermis* from New Zealand is close to the middle of the posterior part (Poinar et al., 2002), in contrast with the position observed in the Chilean mermithids, where it has a spur in the middle of the end part. On the other hand, even if Poinar et al. (2002) analyzed adult specimens and confirmed that *Thaumamermis* may be restricted to crustacean hosts, the *Gammarimermis* would still be an exception, that is restricted to amphipods. However, there is still status in question, such as *Mermi gammari* (von Linstow, 1892), which was described based on juvenile characters, could not be assigned to any known genus, and was placed in the genus *Agamomermis*. This problem has also been reported in mermithids of isopods and insects, especially when juvenile specimens are analyzed, indicating this taxonomic gap and that specimens could belong to multiple genera (Nickle, 1972; Stubbins et al., 2016; Tobias et al., 2017; Yoshino and Waki, 2021).

Among the genera of the family Mermithidae, *Thaumamermis* has been reported in terrestrial isopods (*T. cosgrovei*) and marine amphipods (*T. zealandica*) (Poinar, 1981; Poinar et al., 2002). This genus has an unusual geographic distribution, being known from California (*T. cosgrovei*) and New Zealand (*T. zealanadica*), at localities by the Pacific Ocean, such as Chile. However, our phylogenetic analysis indicated that the Chilean Mermithidae gen. sp. indet., a parasite of *O. tuberculata* from Ramuntcho and Curiñanco beaches, is closely related to mermithids (*Hexamermis agrotis*) that infect insects, such as the order Coleoptera, and to *Agamermis* sp. that infects insects belonging to the order Hemiptera. Therefore, our study shows that *Thaumamermis* is not the only mermithid genus infecting amphipods (Stubbins et al., 2016; Tobias et al., 2017).

Our molecular analysis showed that Mermithidae gen. sp. indet. has an extremely low genetic variation, resulting no variation was detected among individuals, a strong indicative that we are dealing with a single species. Our results are in accordance with several studies that observed low genetic variation in populations of mermithids (Tang and Hyman, 2007; Stubbins et al., 2016; Tobias et al., 2017). Low levels of genetic diversity could be a characteristic of mermithid populations (Poinar et al., 2002; Tang and Hyman, 2007; Yoshino and Waki, 2021). Factors such as low mutation rates or recent population expansions could explain this lack of sequence variation (e.g., Lessa et al., 2010; Goulding and Cohen, 2014).

Differences among the phylogenetic relationships obtained here and those of previous studies are difficult to interpret due to the differences in taxonomic sampling between analyses and possible incongruencies between gene and species trees (e.g., Pamilo and Nei, 1988), as well as possible misidentified specimens. As such, it is clear that further systematic studies are needed to have a good understanding of the evolutionary history of the family Mermithidae. Therefore, we will not discuss here the lack of monophyly of some of the analyzed genera (see Fig. 2B) and the potential classificatory consequences of those phylogenetic patterns. Future research needs increased collection efforts, integrating morphological and molecular data in order to obtain a robust phylogeny that would support a more stable classification for the family Mermithidae. Similarly, increased field-based observations are needed to clarify the life cycle including host identity and to find new species.

## Funding

SMR is supported by ANID-SIA #85220111. GD is supported by 10.13039/501100002850FONDECYT 1221115.

## Data availability

Data will be made available on request.

## Declaration of competing interest

The authors declare that they have no known competing interest.
