# Supplementary material for: First report of a member of the family Mermithidae parasitizing the sandhopper Orchestoidea tuberculata (Amphipoda, Talitridae) in Chile
Source: Int J Parasitol Parasites Wildl. 2023 Oct 31;22:229–33. doi: 10.1016/j.ijppaw.2023.10.011 (PMC10652143; doi:10.1016/j.ijppaw.2023.10.011)
Supplement: Multimedia component 1 [file mmc1.docx]

References Tabla S1

1. Belaich MN, Buldain D, Ghiringhelli PD, Hyman B, Micieli MV, Achinelly MF. 2015. Nucleotide sequence differentiation of Argentine isolates of the mosquito parasitic nematode *Strelkovimermis spiculatus* (Nematoda: Mermithidae). Journal of Vector Ecology, 40, 415–418.

2. Crainey JL, Wilson MD, Post RJ. 2009. An 18S ribosomal DNA barcode for the study of *Isomermis lairdi*, a parasite pf the blackfly *Simulium damnosum s.l.* Medical and Veterinary Entomology, 23, 238–244.

3. Holterman M, van der Wurff A, van den Elsen S, van Megen H, Bongers T, Holovachov O, Bakker J, Helder J. 2006. Phylum-wide analysis of SSU rDNA reveals deep phylogenetic relationships among nematodes and accelerated evolution toward brown clades. Molecular Biology and Evolution, 23, 1792–1800.

4. Iryu T, Tanaka R, Yoshiga T. 2020. Mermithid nematodes isolated from the shield bug *Parastrachia japonensis*. Nematological Research, 50, 1–7.

5. Kobylinski K, Sylla M, Black IV W, Foy BD. 2012. Mermithid nematodes found in adult *Anopheles* from southeastern Senegal. Parasites & Vectors, 5, 131.

6. Mazza G, Paoli F, Strangi A, Torrini G, Marianelli L, Sabbatini Peverieri G, Binazzi F, et al. 2017. *Hexamermis* *papillae* n. sp. (Nematoda: Mermithidae) parasitizing the Japanese beetle *Popillia japonica* Newman (Coleoptera: Scarabaeidae) in Italy. Systematic Parasitology, 94, 915–926.

7. Mullin PG. 2004. Toward a phylogeny for Dorylaimida (Nematoda): Systematic studies in the subclass Dorylaimia. PhD Thesis, ETD collection for University of Nebraska - Lincoln. AAI3137861. <https://digitalcommons.unl.edu/dissertations/AAI3137861>

8. Pernin A, Zanzani S, Mereghetti V, Manfredi MT, Lozzia G, Montagna M. 2015. First record of a mermithid nematode in the leaf beetles *Galeruca laticollis* (Coleoptera: Chrysomelidae). Russian Journal of Nematology, 23, 73–75.

8. Poinar GO Jr, Porter SD, Tang S, Hyman BC. 2007. *Allomermis solenopsi* n. sp. (Nematoda: Mermithidae) para- sitising the fire ant *Solenopsis invicta* Buren (Hymenoptera: Formicidae) in Argentina. Systematic Parasitology, 68, 115–128

9. Powers TO, Mullin PG, Harris TS, Sutton LA, Higgins RS. 2005. Incorporation molecular identification of *Meloidogyne* spp. into a large-scale regional nematode survey. Journal of Nematology, 37, 226–235.

10. Presswell B, Evans S, Poulin, R, Jorge F. 2015. Morphological and molecular characterization of *Mermis nigrescens* Dujardin, 1842 (Nematoda: Mermithidae) parasitizing the introduced European earwig (Dermaptera: Forficulidae) in New Zealand. Journal of Helminthology, 89, 267–276.

11. Sato T, Watanabe K, Tamotsu S, Ichikawa A, Schmidt-Rhaesa A. 2012. Diversity of nematomorph and cohabiting nematode parasites in riparian ecosystems around the Kii Peninsula, Japan. Canadian Journal of Zoology, 90, 829–838.

12. St-Onge M, LaRue B, Charpentier G. 2008. A molecular revision of the taxonomic status of mermithid parasites of black flies of Quebec (Canada). Journal of Invertebrate Pathology, 98, 299–306.

13. Stubbins FL, Agudelo P, Reay-Jones FPF, Greene JK. 2016. *Agamermis* (Nematoda: Mermithidae) infection in South Carolina agricultural pests. Journal of Nematology, 48, 290–296.

14. Sun, LF, He X, Cao F, Bandason E, Shapiro-llan D, Ruan W, Wu S. 2020. First report of *Ovomermis sinensis* (Nematoda: Mermithidae) parasitizing fall armyworm *Spodoptera frigiperda* (Lepidoptera: Noctuidae) in China. Journal of Nematology, 52, 1–7.

15. Tang S, Hyman CC. 2007. Mitochondrial genome haplotype hypervariation within the isopod parasitic nematode *Thaumamermis cosgrovei.* Genetics, 176, 1139–1150.

16. Tobias ZJC, Jorge F, Poulin R. 2017. Life at the beach: comparative phylogeography of a sandhopper and its nematode parasite reveals extreme lack of parasite mtDNA variation. Biological Journal of the Linnean Society, 122, 113–132.16. Tobias

17. Vandergast AG, Roderick GK. 2003. Mermithid parasitism of Hawaiian *Tetragnatha* spiders in a fragmented landscape. Journal of Invertebrate Pathology, 84, 128–136.

18. van Megen H, van del Elsen S, Holterman M, Karssen G, Mooyman P, Bongers T, Holovachov O, Bakker J, Helder J. 2009. A phylogenetic tree of nematodes based on about 1200 full-length small subunit ribosomal DNA sequences. Nematology, 11, 927–950

19. Villemant C, Zuccon D, Rome Q, Muller F, Poinar Jr GO, Justine JL. 2015. Can parasites halt the invader? Mermithid nematodes parasitizing the yellow-legged Asian hornet in France. PeerJ, 3, e947.

20. Yeates GW, Buckley TR. 2009. First records of mermithid nematodes (Nematoda: Mermithidae) parasitizing stick insects (Insecta: Phasmatodea). New Zealand Journal of Zoology, 36: 35–39.

21. Yoshino H, Waki T. 2021. First report on Mermithidae (Mermithida) infection in *Ligidium* sp. (Isopoda, Ligiidae). Parasitology International, 82, 102304

22. Watanabe S, Tsunashima A, Itoyama K, Shinya R. 2021. Survey of mermithid nematodes (Mermithida: Mermithidae) infecting fruit-piercing stink bugs (Hemiptera: Pentatomidae) in Japan. Applied Entomology and Zoology, 56, 27–39.
