# Supplementary material for: First report of a member of the family Mermithidae parasitizing the sandhopper Orchestoidea tuberculata (Amphipoda, Talitridae) in Chile
Source: Int J Parasitol Parasites Wildl. 2023 Oct 31;22:229–33. doi: 10.1016/j.ijppaw.2023.10.011 (PMC10652143; doi:10.1016/j.ijppaw.2023.10.011)
Supplement: Multimedia component 2 [file mmc2.docx]

**Table S1.-** Species of mermithids, their hosts, location, and Genbank accession number used for phylogenetic analysis based on the cox1 and 18S gene. (–) Location not indicated.

| Species | Code | Host | Location | COI | 18S | References |
| --- | --- | --- | --- | --- | --- | --- |
| Agamermis sp. | – | Pentatomidae | – | DQ665656 | – | Unpublished data |
| Agamermis changshaensis | – | Nephepterix leucophaea | – | ­– | DQ628908 | Unpublished data |
| Agamermis xianyangensis | – | ­Black flies | – | – | EF617352 | Unpublished data |
| Agamermis sp. | BH-2006 | _ | _ | _ | DQ665653 | Unpublished data |
| Agamermis sp. | FLS-2016 | Megacopta cribaria | – | – | KX173336 | Sttubins et al. [13] |
| Allomermis solenopsii | – | Solenopsis invicta | – | – | DQ533953 | Poinar et al. [8] |
| Amphimermis sp. | A-2007 | Orthoptera | ­– | – | EF617354 | Unpublished data |
| Gastromermis viridis | – | Black flies | Canada | – | EU792502 | St-Onge et al. [12] |
| Gastromermis sp. | AS | Diptera | – | – | AY146543 | Mullin et al. [7] |
| Gastrmermis sp. | BH-2006 | – | – | – | DQ533954 | Unpublished data |
| Heleidomermis sp. | BH-2006 | Culicoides | ­– | – | DQ533955 | Unpublished data |
| Hexamermis agrotis | – | Agrotis segetum | – | EF368011 | DQ530350 | Unpublished data |
| Hexamermis albicans | – | Succinea putriis | Hungary | – | KF732650 | Unpublished data |
| Hexamermis popilliae | – | Popillia japonica | Italy | – | MK040823 | Mazza et al. [6] |
| Hexamermis sp. | RS-2021 | Glaucias subpunctatus | _ | _ | LC661691 | Watanabe et al. [21] |
| Isomermis lairdi | – | Simulium squamosum | – | ­– | FN400898 | Crainey et al. [2] |
| Isomermis wisconsinensis | – | Black flies | Canada | – | EU792503 | St-Onge et al. [12] |
| Limnomermis sp. | JH-2014 | – | – | – | KJ636371 | Van Megen et al. [18] |
| Mermis nigrescens | – | Forficula auricularia | New Zealand | – | KF583883 | Presswell et al. [19] |
| Mermis sp. | 9 mile 430LP2-53 | – | USA | – | AY919185 | Powers et al. [9] |
| Mermis sp. | K-06 | Culicoides obsoletus | Czech Republic | – | FJ973464 | Unpublished data |
| Mesomermis camdenensis | – | Black flies | Canada | – | EU792504 | St-Onge et al. [12] |
| Mesomermis flumenalis | – | Black flies | Canada | _ | EU792505 | St-Onge et al. [12] |
| Octomyomermis huazhongensis | – | Diptera | China | – | EF617353 | Unpublished data |
| Ovomermis sinensis | ­– | Spodoptera frugiperda | China | – | KU177046 | Sun et al. [14] |
| Pheromermis sp. | MNHNJL50 | Vespa velutina | France | – | KR029620 | Villemant et al. [19] |
| Romanomermis culicivorax | – | Culicidae | – | ­– | DQ418791 | Unpublished data |
| Romanomermis iyengar | – | Culicidae | – | – | JX021620 | Unpublished data |
| Romanomermis sichuanensis | – | Culicidae | – | – | Ef612769 | Unpublished data |
| Romanomermis wuchangensis | – | Culicidae | – | – | DQ520878 | Unpublished data |
| Strelkovimermis spiculatus | – | Aedes albifasciatus | – | DQ520860 | KP270704 | Belaich et al. [1] |
| Thaumamermis zealandica | – | Bellorchestia quoyana | New Zealand | KY264161 | KY264164 | Tobias et al. [16] |
| Thaumamermis cosgrovei | – | Armadillidium vulgare | Botanic Garden. U. California, USA | NC008046 | – | Tang & Hyman [15] |
| Thaumamermis cosgrovei | – | Armadillidium vulgare | Botanic Garden. U. California, USA | DQ520857 | – | Tang & Hyman, [15] |
| Thaumamermis cosgrovei | – | Armadillidium vulgare | Botanic Garden. U. California, USA | DQ520858 | – | Tang & Hyman, [15] |
| Thaumamermis cosgrovei | – | – | – | – | DQ665655 | Unpublished data |
| Mermithidae sp. | – | – | Japan | – | LC661693 | Watanabe et al. [22] |
| Mermithidae sp. | 1KCK-2013 | Anopheles gambiae | Senegal | – | KC243312 | Kobylinski et al. [5] |
| Mermithidae sp. | A-AV-2003 | Spiders | Hawaii | – | AY374415 | Vandergast & Roderick, [17] |
| Mermithidae sp. | B-AV-2003 | Insects | Hawaii | – | AY374416 | Vandergast & Roderick, [17] |
| Mermithidae sp. | JH-2004 | – | – | – | AY284743 | Holterman et al. [3] |
| Mermithidae sp. | Kagoshima | Parastrachia japonensis | Japan | – | LG512371 | Iryu et al. [4] |
| Mermithidae sp. | KW-2011-M1 | – | Japan | – | AB647224 | Sato et al. [11] |
| Mermithidae sp. | MM-2014 | Galeruca laticollis | Italy |  | HG970633 | Pernin et al. [8] |
| Mermithidae sp. | Saga | Plautia stali | Japan | – | LC512372 | Iryu et al. [4] |
| Mermithidae sp. | Shinbayashi-2017A | Ligidium sp. | Japan | – | LC596451 | Yoshino & Waki, [21] |
| Mermithidae sp. | TB-2009 | Stick insects | New Zealand | – | FJ605514 | Yeates & Buckley, [20] |
